# Impact of climate change on foodborne infections and intoxications

**DOI:** 10.25646/11403

**Published:** 2023-06-01

**Authors:** Jessica Dietrich, Jens-Andre Hammerl, Annette Johne, Oliver Kappenstein, Christopher Loeffler, Karsten Nöckler, Bettina Rosner, Astrid Spielmeyer, Istvan Szabo, Martin H. Richter

**Affiliations:** 1 German Federal Institute for Risk Assessment Department for Safety in the Food Chain Berlin, Germany; 2 German Federal Institute for Risk Assessment Department for Biological Safety Berlin, Germany; 3 Robert Koch Institute Department of Infectious Disease Epidemiology Berlin, Germany

**Keywords:** CAMPYLOBACTER, SALMONELLA, VIBRIO, CRYPTOSPORIDIUM, GIARDIA, MARINE BIOTOXINS, ONE HEALTH

## Abstract

**Background:**

Temperature, precipitation, and humidity are important factors that can influence the spread, reproduction, and survival of pathogens. Climate change affects these factors, resulting in higher air and water temperatures, increased precipitation, or water scarcity. Climate change may thus have an increasing impact on many infectious diseases.

**Methods:**

The present review considers those foodborne pathogens and toxins in animal and plant foods that are most relevant in Germany, on the basis of a selective literature review: the bacterial pathogens of the genera *Salmonella, Campylobacter* and *Vibrio*, parasites of the genera *Cryptosporidium* and *Giardia*, and marine biotoxins.

**Results:**

As climate change continues to progress, all infections and intoxications discussed here can be expected to increase in Germany.

**Conclusions:**

The expected increase in foodborne infections and intoxications presents a growing public health risk in Germany.

## 1. Introduction

Temperature, precipitation, humidity, and soil properties are important environmental factors that influence the spread and survival of zoonotic pathogens. Changes in these environmental factors as a result of climate change, such as permanently elevated ambient temperatures, increasing precipitation, but also water scarcity, may contribute to the spread and survival of pathogens. Climate change may thus exert an increasing influence on more than half of all infectious diseases [1]. This is not only true for already existing, i. e. endemic, infectious agents; climatic changes also favour the establishment of novel infectious agents (emergence) as well as the return of pathogens suppressed in the past (re-emergence). For example, in the future, agriculture may have to rely more frequently on treated wastewater due to water scarcity, which will be exacerbated by climate change. This poses a number of risks to food safety, including contamination of irrigated produce by various types of pathogens [2].

This review addresses hazards to human health posed by the most important foodborne bacteria, parasites and marine biotoxins in Germany and presents recommendations for reducing the risks. For example, the risk of all infections discussed here can be reduced by observing good hygiene during food preparation (kitchen hygiene) and adherence to cold chains.

Since viruses play a minor role in the impact of climatic changes on foodborne infections, they are not further discussed in this article. Their importance in the context of climate change is discussed in detail in other parts of this status report, e. g. in connection with vector-borne infectious diseases (Beermann et al. [3]).

## 2. Bacteria

### 2.1 Campylobacter

Bacteria of the genus *Campylobacter* (*C.*) cause an intestinal infection typically associated with abdominal pain and watery, occasionally bloody diarrhoea. Rare complications may include joint inflammation and Guillain-Barré syndrome, a nerve disease associated with paralysis. Even a low infectious dose of ≥500 germs can trigger a *Campylobacter* infection*.* Approximately 50,000 to 70,000 cases are reported to the Robert Koch Institute (RKI, Germany’s national public health institute) annually. For years, *Campylobacter* enteritis has been the most common bacterial diarrhoeal disease in Germany that has a mandatory notification requirement as per the German Protection against Infection Act (IfSG). The most important *Campylobacter* species pathogenic to humans are *C. jejuni* and *C. coli*. Transmission to humans occurs primarily via contaminated food of animal origin. In particular, consumption of contaminated chicken meat is a significant risk factor for *Campylobacter* infection [4–6]. Transmission to humans is also possible via other foods like non-pasteurised milk. Unlike *Salmonella*, *Campylobacter* is considered unlikely to multiply in food because of its microbiological characteristics (growth under microaerobic conditions, i. e. reduced oxygen concentration in the atmosphere, and at temperatures of 30°C to 42°C) [7]. *Campylobacter* infections via contaminated drinking water or bathing water contaminated with animal faeces have also been described [8, 9].

#### Climate influence on *Campylobacter* infections

*Campylobacter* enteritis infections typically follow a seasonal course, with the highest number of cases occurring in the summer months from July to September, including non-travel-associated infections. With progressive warming as a result of climate change and the associated prolonged warm periods, an increase in *Campylobacter* cases in humans is therefore expected.

It is conceivable that during the summer months, increased temperatures lead to higher *Campylobacter* prevalence in poultry flocks and thus higher exposure of consumers via consumption of poultry meat, although data on this are not consistent [10–14].

Altered consumption and recreational behaviours during the summer months have an important indirect effect on the increase in human infections, if they are associated with increased exposure, thus promoting infections, such as more frequent barbecuing of poultry and other meats, or swimming in surface waters [14, 15].

An association of human *Campylobacter* infections has been linked not only to temperature [16, 17] but also to the amount of precipitation in the days preceding the onset of illness [18, 19]. An increase in *Campylobacter* infections and *Campylobacter* enteritis outbreaks has been observed after heavy rains and flooding, likely due to increased exposure via faecal-contaminated surface water or contaminated drinking water [9, 20]. In contrast, *Campylobacter* cases are expected to decrease after periods of drought [18, 19].

With increasing water scarcity as a result of climate change, it is conceivable that treated wastewater, which may be contaminated with pathogens from animal or human faeces, will be used more frequently for irrigation of plant-based foods [21]. Increasing contamination of plantbased foods with gastrointestinal pathogens, including *Campylobacter*, would be expected via this route, thus compromising food safety [21]. European Union (EU) Regulation 2020/741 is intended to counteract this and regulates minimum standards for the use of treated wastewater in agricultural irrigation (Info box).

In a model calculation, the effect of increasing temperature and precipitation on the number of *Campylobacter* cases in Scandinavia was estimated. A doubling of *Campylobacter* cases by the year 2080 was predicted for Denmark, Finland, Norway, and Sweden [19]. Further scientific studies are needed to better understand the multifactorial, direct and indirect relationships between climatic changes and *Campylobacter* disease cases.

**Info box EU regulation on water reuse**

Climatic changes are increasing the pressure on water resources in Germany and Europe. To counter this pressure, Regulation (EU) 2020/741 established minimum requirements for the use of treated wastewater in agricultural irrigation. It came into effect on June 26, 2020, and will apply to all member states of the European Union from June 26, 2023.

The regulation is intended to reduce water scarcity in the European Union as a result of climate change by reusing water for agricultural irrigation and to facilitate implementation by the member states with uniform requirements. The aim is to achieve a high level of protection for the environment and for human and animal health, and to promote the circular economy [22]. The regulation on minimum requirements for water reuse is limited to agricultural irrigation. In addition to uniform minimum requirements for water quality and monitoring, risk management and provisions for data transparency are the main elements of the regulation [22]. In this context, the German Federal Institute for Risk Assessment (BfR) published opinions on possible risks associated with the reuse of treated wastewater for irrigation of edible crops [23–25]. Even wastewater that has already been treated may still contain parasites, bacteria and viruses in pathogenic concentrations. Therefore, particular care should be taken to ensure that plant parts that are usually consumed raw do not come into direct contact with irrigation water or, if this cannot be safely avoided, should continue to be irrigated with potable water. Persons belonging to risk groups are advised against consumption of such raw foods.

### 2.2 *Salmonella*

The bacteria of the genus *Salmonella* are zoonotic agents that can be transmitted directly or indirectly between humans and animals. They are widely distributed in nature. The main reservoir for *Salmonella* is warm-blooded animals, including livestock and wildlife. *Salmonella* also occurs in cold-blooded animals, such as reptiles or insects, which can act as vectors and transmit *Salmonella* to warm-blooded animals or humans. Although most salmonellae do not usually cause symptoms in animals, they can cause mild to severe health problems in humans. Human salmonellosis is often accompanied by fever, nausea, vomiting, abdominal pain, and headache, but signs of illness may be completely absent.

In Germany, salmonellosis is the second most frequently reported bacterial foodborne infection in humans after campylobacteriosis; *Salmonella (S.) enterica* is an important cause of foodborne outbreaks. Among these, *S*. Enteritidis and *S*. Typhimurium are the most common serovars, together accounting for approximately 75% of all reported salmonellosis cases [26]. Poultry is presumed the most important source of *Salmonella* infections in humans. In particular, eggs and egg products play a predominant role. Pork and pork products represent another important source [27, 28]. Increasingly, *Salmonella* infections are also reported to be associated with the consumption of foods of non-animal origin [27, 29]. Among these, raw leafy vegetables, onion and stem vegetables, tomatoes, and melons were the most commonly affected products. Contamination of these products with *Salmonella* can occur both before harvest (through faecal matter, irrigation water, dust, insects, etc.) and after harvest (through harvesting equipment, transport containers, insects, dust, rinsing water, ice, transport vehicles, processing equipment) [30].

#### Climate influence on *Salmonella* infections

In Europe, most salmonellosis cases are reported during the summer months [28]. The incidence of *Salmonella* is often lower in northern countries than in countries located in warmer climates.

Ambient temperature can influence the development of *Salmonella* at different stages of the food chain: e. g. bacterial contamination during raw food production, transport, and improper storage [31]. The optimal temperature for *Salmonella* growth is between 35°C and 37°C; below 15°C, growth of *Salmonella* is greatly reduced. Consequently, *Salmonella* multiply faster at higher temperatures. The significant correlation between outdoor temperatures and outbreaks caused by *Salmonella* has been known for some time. Studies have reported the increase of salmonellosis, as well as other bacterial enteric diseases, with increasing temperatures [32]. According to Zhang et al. [31], an increase of 8.8% in the number of weekly cases can be expected for a 1°C increase in the mean weekly maximum temperature. With a 1°C increase in the mean weekly minimum temperature, a 5.8% increase in the weekly number of cases can be expected.

Temperature can affect the transmission of *Salmonella* to humans through several pathways; by directly affecting the multiplication of *Salmonella* and by indirectly affecting eating habits during hot days. The favoured growth of *Salmonella* at higher temperatures leads to higher concentrations of *Salmonella* in contaminated foods during the warmer months. Among other things, this has to do with poor food preparation and refrigeration during barbecues or picnics, which are also more common during these months. Elevated ambient temperatures increase the risk of cold chain disruption, which can have a significant impact on the microbiological status of food.

Zhang et al. [33] found a strong correlation between drinking water quality, precipitation and gastroenteritis, where maximum and minimum temperatures, relative humidity and precipitation correlated positively with the number of salmonellosis cases.

In areas with increased rainfall, water quality could deteriorate. Heavy rains can increase run-off in rivers and lakes, washing sediment, pollutants, refuse, animal waste, and other materials into water supplies. Severe flooding can inundate wastewater treatment plants. This can lead to contamination of human, animal, and farm environments by bacteria in human sewage that are not only infectious, but can also be resistant to antimicrobial agents. Another article in this status report discusses this issue separately in the context of climate change impacts on health due to extreme weather events (Butsch et al. [34]).

### 2.3 *Vibrio*

*Vibrio* are environmental bacteria that colonise saline and brackish water bodies as well as wetlands worldwide, but can also occur in the microbial flora of aquatic animals. For humans, water contact and ingestion of *Vibrio (V.)*-containing seafood (fish, marine animals) can be problematic. As bacterial contaminants, *V. cholerae*, *V. vulnificus*, and *V. parahaemolyticus* in particular can cause infections [35, 36]. The significance of each species for humans varies regionally and is linked to area-specific factors, such as the salinity of water bodies, air and water temperatures, and fluctuations to which they are subject [37]. The genus *Vibrio* includes more than 135 species [38], three of which are largely causative of intestinal (e. g. gastroenteritis) or extraintestinal infections (e. g. middle ear infection) [35]. *V. cholerae* serotypes O1/O139, which produce the cholera toxin, are significant worldwide, as they can lead to pandemic cholera outbreaks in tropical and subtropical regions with poor water hygiene. In Europe, the only endemic *Vibrio* types are *V. cholerae* non-O1/non-O139 serogroups and other *Vibrio species*, collectively called non-cholera *Vibrio* (NCV). *V. cholerae* non-O1/non-O139 can lead to self-limiting infections with moderate diarrhoeal symptoms, but are rarely associated with food consumption. Gastrointestinal illness in Europe is mainly associated with haemolysin (*trh/ tdh*)-encoding *V. parahaemolyticus* isolates and consumption of raw or undercooked seafood. *V. vulnificus*, on the other hand, plays an important role in severe wound infections, which is discussed in another article in this status report that looks more closely at effects of climate change on waterborne infections and intoxications (Dupke et al. [39]). This species is common in coastal waters of moderate salinity worldwide and has been associated with fatal infections from consumption of contaminated oysters, with mortality rates for primary septicaemia (blood poisoning) sometimes exceeding 50% [37]. Accurate information on foodborne *Vibrio* infections is not yet available, as pathogen detection in diarrhoeal diseases is often not part of risk-based diagnostics, and a mandatory notification requirement for human *Vibrio* infections does not exist throughout Europe. In Germany, only isolated cases of gastrointestinal NCV infections have been recorded since the introduction of mandatory notification according to the IfSG in 2020, which may indicate either low exposure to *Vibrio*-containing products or that a large proportion of the illnesses are not detected and thus not reported [40].

The importance of seafood has increased due to its high protein, vitamin and mineral content. For many years, the annual per capita consumption in Germany was covered by wild catch, but now increasingly comes from aquacultures. Usually, seafood is offered in processed form (e. g. heated, marinated, smoked) and should thus contain no or hardly any *Vibrio*. However, raw and insufficiently heated products pose a risk [40]. Mussels and oysters in particular are predestined to enrich even small amounts of *Vibrio* due to their way of filtering of nutrients from the water. Thus, they pose a health risk, especially for people with weakened immune systems or pre-existing conditions.

In mussels and oysters, *V. alginolyticus* (sometimes associated with middle ear infections in humans) and *V. parahaemolyticus* are frequently detected, and rarely *V. cholerae* non-O1/non-O139 and *V. vulnificus* have been reported, with *V. alginolyticus* occurring year-round and the other species occurring exclusively during the warm summer and fall months [41]. Within Europe, several countries (e. g. Spain, France, United Kingdom) cultivate mussels and oysters, some of which are heavily contaminated with toxigenic *Vibrio* (up to 25% toxigenic *V. parahaemolyticus* ) [40].

There are hardly any reliable data available on the probability of occurrence of a health impairment after exposure to *Vibrio* species pathogenic to humans*.* In principle, the risk of health impairment correlates with the amount of ingested pathogenic *Vibrio* and is also dependent on the respective species. In addition, the probability of occurrence of a health impairment is increased in vulnerable groups of people (YOPI: young, old, pregnant, immunocompromised people).

#### Climate influence on *Vibrio* infections

Compared to the rest of the world, foodborne *Vibrio* infections have been rare in Europe so far, which may change in the future in the course of climate change. In general, water temperatures above 12°C and low or moderate salinity (1–25 g/L) have a favourable effect on the occurrence of *Vibrio* spp. [36]. Optimal growth conditions already occur in Europe during the summer months on the Atlantic coast and in inland seas [42]. The occurrence of *Vibrio* spp*.* is favoured by global warming and the increase of heatwaves and may lead to the spread of endemic *Vibrio* and possibly also to the establishment of new pathotypes in Europe, so that the human infection incidence may increase in the future, especially in coastal areas and estuaries [42, 43]. The continuous increase in water temperature will lead to an amplification of *Vibrio* contamination in European seafood catching, harvesting, and farming areas, and will also expand beyond the summer and autumn months. Currently, the occurrence of pathogenic *Vibrio* is low in bodies of water with fluctuating temperatures, such as the North Sea and the Baltic Sea, whereas toxigenic NCV are increasingly detected in waters with consistently warm temperatures, from which seafood is partly imported [41]. In seafood, the occurrence of species pathogenic to humans correlates directly with water temperatures as well [36, 40].

Minimisation of risk to humans, especially in relation to contact with and consumption of seafood, is possible by avoiding exposure to pathogenic species, e. g. through appropriate product processing strategies, thermal treatments, adherence to hygienic measures, a strict cold chain, and good and rapid monitoring immediately after capture, harvest, or import [40].

## 3. Parasites

Climate change can also influence infections by parasites, especially if these are characterised by a significant environmental presence and high environmental stability. This applies in particular to protozoa, unicellular organisms that live as parasites. Even if profound knowledge is lacking, recent research data (German Federal Institute for Risk Assessment (BfR); data not published) indicate that a changing climate also has a direct impact on the prevalence and virulence of these pathogens, which are already very stable in the environment. A risk of transmission to humans through food is usually associated with those foods that are consumed raw or inadequately cooked. Food contamination can occur via vectors (e. g. insects, mammals including those providing food to humans) but also via contaminated irrigation systems. In addition, food can be affected by cross-contamination, such as when hygiene is poor during food preparation.

### 3.1 Cryptosporidia

More than 40 different species of the protozoan genus *Cryptosporidium* have been described to date, which can infect a large number of different animal species and humans. Here, we focus on the species *Cryptosporidium (C.) parvum* and *C. hominis*, as they are responsible for the largest proportion of human infections [44]. *C. hominis* occurs almost exclusively in humans, while the primary hosts for *C. parvum* are cattle, horses, goats, and sheep, and, to a lesser extent, dogs, cats, and birds [45]. Infection occurs via the faecal-oral route by ingestion of the infective developmental stages (oocysts); transmissions from animal to animal, human to human, animal to human, and vice versa are possible [46]. However, infection can also occur through ingestion of contaminated water (e. g. swimming pool, river, lake, or spring water). Another source of infection can be plant-based foods that have been contaminated with infested water.

Cryptosporidiosis is one of the most common diarrhoeal diseases associated with the ingestion of contaminated water. In 2013, an outbreak with 167 cases was recorded in Germany in association with flooding after a heavy rain event [47]. Foodborne illnesses have also been described in Europe, which have been attributed to the consumption of lettuce [24].

In Germany, there is a mandatory notification requirement for detection of cryptosporidia in connection with an illness in accordance with the IfSG. Between 900 and 2,000 cases are reported to the RKI annually; in Europe, there are 8,000 to 14,000 cases annually. Data from the European Centre for Disease Prevention and Control (ECDC) show that cryptosporidiosis in Europe has seasonal increases in late spring and late summer to early autumn, respectively [48].

Young children, people with weakened immune systems, travellers to developing countries, and people who drink untreated water are at increased risk of infection.

Infection may be asymptomatic or symptomatic. Symptoms described include prolonged watery diarrhoea with weight loss, severe abdominal pain or cramps, nausea, vomiting, and headaches [49]. After infection, further complications (e. g. inflammation of the pancreas, appendicitis, impairment of the lungs) and even death may occur. After the symptoms have subsided, the oocysts continue to be excreted in the stool for many weeks. The infectious dose of cryptosporidia is low, ranging from 10 to 1,000 oocysts, depending on the species [50]. However, it is suspected that one oocyst may be sufficient for infection in humans [51].

### 3.2 *Giardia*

Parasites of the protozoan genus *Giardia* can also be affected by climate change. *Giardia* are currently classified into eight species [52]. However, the species *Giardia (G.) duodenalis* (synonyms: *G. lamblia*, *G. intestinalis*) is the only species of this genus that can infect humans as well as numerous mammalian species. *G. duodenalis* is distributed worldwide; humans are considered the main reservoir. Infection occurs faecal-orally by ingestion of the infectious developmental stages of the parasite (1 to 10 cysts are sufficient) with contaminated tap water or untreated fresh water from lakes or streams. However, *Giardia* cysts can also be transmitted by eating contaminated food or by close contact with infected persons or animals [53].

Giardiasis caused by *G. duodenalis* is one of the most common intestinal parasitoses in humans worldwide [54]. Prevalence rates are significantly higher in developing countries than in developed countries. For example, 3,296 travel-associated cases of the disease were reported to the RKI in 2019; the most frequently cited country of infection was India [26]. However, as warming due to climate change continues, increased local infections will also be expected. Infants and young children, older persons, travellers, and immunocompromised persons are among the high-risk groups [55]. In more than 50% of cases, the disease is asymptomatic, but the course can be severe in infants and older persons [56].

In Germany, detection of this pathogen in connection with an illness is notifiable.

Globally, *G. duodenalis* is estimated to cause 28.2 million cases of diarrhoeal disease per year due to food contamination [52]. The European Food Safety Authority (EFSA) recorded 14 foodborne outbreaks and 3 outbreaks due to water consumption in Europe in 2019 [57]. Data on foodborne outbreaks in Germany are not yet available.

### 3.3 Climate influence on parasites

*Cryptosporidium* and *Giardia* can remain infectious for a long period of time and cause disease, especially after consumption of raw contaminated food.

Their high stability against environmental influences, in particular the long survivability in aqueous environments, suggests that these parasites could appear more frequently as pathogens in the future. Extreme weather such as heavy rainfall and flooding, which are also expected to increase in our latitudes as a result of climate change, increase the risk of infectious oocysts/cysts entering bodies of water, as well as the risk of contamination of plant-based foods. The risk for humans can be minimised by good kitchen hygiene.

## 4. Biogenic toxins of marine origin

More than 20% of the protein requirements of over 3 billion people worldwide are met by seafood. Consumption of fish as food has increased by 3.1% annually over the last 50 years [58]. Climate change is increasingly causing warming, acidification, and oxygen depletion of the oceans, altered water salinity, and sea level rise. The temperature of the oceans’ surface water has increased greatly, as more than 90% of the global temperature increase has been absorbed by the oceans [59]. These changes are affecting marine biodiversity, which in turn can significantly impact the availability of food, animal protein, and essential micronutrients for billions of people around the world [60, 61].

The above factors can also affect the abundance and composition of marine phytoplankton. Planktonic microalgae form the basis of the aquatic food web, but can have harmful effects if they occur in large abundance. The phenomenon of sudden, explosive growth of algae is called an algal bloom. When these algal blooms have negative consequences, such as creating hypoxic or anoxic conditions (lack of oxygen or complete absence of oxygen) or producing toxins, they are referred to as harmful algal blooms (HABs). HABs have the potential to disrupt fish communities and food webs, and can even lead to massive species extinctions [62]. Currently, approximately 300 microalgal species are known to be involved in the formation of such algal blooms. Of these, about 100 species can produce toxins that can lead to specific toxic syndromes in animals and humans.

Marine biotoxins enter the food chain through organisms that filter water or feed on algae, such as shellfish and fish, and thus may pose a threat to human health due to their toxic properties [63, 64]. Furthermore, exposure to toxins produced by marine phytoplankton can occur through inhalation or through the skin, via aerosols that contain toxins and cell fragments and can cause skin irritation and respiratory symptoms [65, 66].

Climate change is altering the geographic distribution of some algal species that may be involved in the formation of HABs. Warm-water species, for example, may be expanding poleward and appearing in areas where they were not previously native. In this context, identifying and studying the geographic distribution of toxin-producing organisms is critical for implementing appropriate preventive and control measures prior to harvest or during the distribution and sale of seafood. This is particularly important because marine biotoxins are not normally detectable by odour, taste, or appearance and are not usually destroyed by cooking, freezing, or other food preparation processes.

In the interests of preventive consumer health protection, maximum levels have been set within the EU for five different toxin groups in live bivalve molluscs. Furthermore, products containing compounds of the ciguatoxin group that can cause mild to severe poisoning (ciguatera) in humans [67] may not be marketed within the EU [68, 69].

## 5. Conclusion and recommendations

Climate change affects various habitats, which can be altered by weather events such as prolonged droughts, temperature increases, and heavy rains. These changes also affect the foods derived from these habitats and micro- organisms or toxins that may be associated with these foods. For example, foods may be more heavily contaminated with pathogens or contain germs that were not previously present in a region. But climate-related events also affect food indirectly. For example, ever-increasing water scarcity means more frequent use of treated wastewater for food irrigation. Because such wastewater may still contain parasites, bacteria, and viruses in disease-causing concentrations, plant parts that are commonly consumed raw should not come into contact with treated wastewater, or should continue to be irrigated with potable water [23–25]. Progressive climate change in Germany is expected to lead to an increase of the infections and intoxications discussed here, making them a growing public health concern.

Our main recommendations for minimising the health risk from foodborne infections and intoxications lie in the area of kitchen hygiene, which should always be applied when preparing food. This includes thorough hand washing and the use of fresh kitchen utensils after handling raw meat and fish, as well as avoidance of cross-contamination, i. e. direct or indirect pathogen transmission from one food to another. Wearing gloves can prevent the entry of pathogens via unnoticed skin lesions. Food safety is also highly dependent on maintaining the cold chain. In addition, most microbiological pathogens can be safely killed by a sufficient heating process; for example, a core temperature of 70°C for at least two minutes must be maintained when preparing seafood. In contrast, biogenic toxins associated with food are largely insensitive to temperature [40, 70, 71].

We also recommend the use of new technologies to track supply chains. Given a globalised food distribution network and the use of different processing and preservation techniques, it can be difficult to track a product’s supply chain to identify potential risks. Technological advances have produced digital solutions for this; knowledge of fish stocks, seafood traceability and supply chain transparency can benefit from innovative approaches. These include blockchain or radio frequency identification device tags for product authentication (including species and catch data), as well as applications of machine learning, data mining, artificial intelligence, and other digital technologies [72]. These methods of digitally-enabled food supply chains can support sustainable development in the food industry, which aims to reward responsible and ethical producers and keep illegal or unethically produced food products out of supply chains [73].

Further research is needed on the links between climatic changes and disease incidence, and on the geographic distribution of toxin-producing organisms.

## Key statement

- The number of infections with germs such as *Salmonella* or *Campylobacter* correlates positively with maximum temperature and precipitation amounts.
- Environmentally associated germs such as waterborne *Vibrio* are increasingly finding their way into food, especially seafood, and causing an increase in infections.
- Rising temperatures and humidity favour fitness and possibly virulence of parasites such as *Cryptosporidium* and *Giardia*.
- An increase in infections due to *Cryptosporidium* and *Giardia* is to be expected.
- Ocean warming is leading to increased growth of toxinproducing algae, whose toxins can enter the food chain via marine animals.
